# Re-evaluating whether bilateral eye movements influence memory retrieval

**DOI:** 10.1371/journal.pone.0227790

**Published:** 2020-01-27

**Authors:** Brady R. T. Roberts, Myra A. Fernandes, Colin M. MacLeod

**Affiliations:** Department of Psychology, University of Waterloo, Waterloo, Ontario, Canada; University of Pittsburgh, UNITED STATES

## Abstract

Several recent studies have reported enhanced memory when retrieval is preceded by repetitive horizontal eye movements, relative to vertical or no eye movements. The reported memory boost has been referred to as the *Saccade-Induced Retrieval Enhancement* (SIRE) effect. Across two experiments, memory performance was compared following repetitive horizontal or vertical eye movements, as well as following a control condition of no eye movements. In Experiment 1, we conceptually replicated Christman and colleagues’ seminal study, finding a statistically significant SIRE effect, albeit with weak Bayesian evidence. We therefore sought to conduct another close extension. In Experiment 2, horizontal and vertical eye movement conditions were manipulated separately, and sample size was increased. No evidence of a SIRE effect was found: Bayesian statistical analyses demonstrated significant evidence for a null effect. Taken together, these experiments suggest that the SIRE effect is inconsistent. The current experiments call into question the generalizability of the SIRE effect and suggest that its presence is very sensitive to experimental design. Future work should further assess the robustness of the effect before exploring related theories or underlying mechanisms.

## Introduction

### Eye movements and memory processes

The discipline of psychology has long been interested in the role that the human visual system plays in everyday cognition. In 1972, Loftus published an article in *Cognitive Psychology* that reported a positive correlation between the number of eye movements that a participant made while viewing a picture and their subsequent memory for that picture [1]. Since then, researchers have linked eye movements to visuo-spatial working memory [2], to external projection of mental imagery [3], and even to direct neuroanatomical connectivity ties to the hippocampus in macaque monkeys [4]. The study of eye movements is now firmly embedded in the study of cognition, and extensions to the memory literature continue to emerge.

In 2003, Christman and colleagues were the first to investigate a new perspective on eye movements and memory—the potential benefit of bilateral eye movements for memory retrieval [5]. They undertook this work because of research in their own lab suggesting that bilateral eye movements can enhance interhemispheric interaction [6], which in turn was theorized to have a role to play in episodic memory processes [7,8]. Their prediction was that repeated horizontal saccadic eye movements, when performed immediately before a memory test, should cause increased interhemispheric interaction and lead to superior retrieval relative to no eye movements at all. That pattern was in fact what they observed. The purpose of the current study was to first perform a close extension of this seminal work by Christman and colleagues in our own lab before ultimately moving forward to test proposed mechanisms underlying the observed memory boost following bilateral eye movements.

Seven years and several articles after Christman et al.’s (2003) work [5], Lyle and Martin published the first study that would refer to their finding as the *Saccade-Induced Retrieval Enhancement* (SIRE) effect [9]. Lyle and Martin argued against Christman et al.’s (2003) account of the reported memory benefit in terms of interhemispheric processes. They employed a letter-matching task both within and across visual fields to provide evidence that the SIRE effect is more likely due to *intrahemispheric* (i.e., within one hemisphere) rather than to interhemispheric (i.e., between hemispheres) activity [9]. Their idea was that detection of letter pairs on the matching task requires intrahemispheric interaction on within-hemisphere trials but mostly interhemispheric processing on across-hemisphere trials. Therefore, the superior performance on within-hemisphere trials that they observed following eye movements suggests increased intra- as opposed to inter- hemispheric interaction [9].

Generally, the SIRE effect has been researched using word stimuli and simple memory retrieval tasks [e.g., 5,8]. Brunyé and colleagues, however, sought to extend the literature to include picture stimuli [10]. Edlin and Lyle (2013) actually went so far as to rename the effect saccade-induced *cognitive* enhancement (SICE) since they have reported benefits of bilateral eye movements extending to executive functioning aspects as well [11]. Further studies have reported broader SIRE benefits in domains including autobiographical memory [12], gist-based false recognition [13], episodic future thinking [14], and childhood amnesia [15].

While there have been several publications reporting significant SIRE effects, there have also been reported failures to replicate findings. Most notably, Matzke et al. (2015) failed to replicate the SIRE effect, consistently finding strong Bayesian evidence for a null [16]. Critically, their method followed a framework of pre-registered adversarial collaboration, which involved two opposing research groups discussing and ultimately agreeing upon specific experimental and statistical methodologies before pre-registering their study on the *Open Science Framework* (OSF). In addition, Samara et al. also failed to find a significant effect when using neutral valence words for stimuli (but did succeed in finding the effect for emotional words) [17]. Further, while some articles have proposed that eye movements can have the side effect of increasing false memory [e.g., 18], a recent direct replication attempt failed [19]. These failed replications are obviously of major concern and served as a main motivation for attempting to conceptually replicate the SIRE effect in our lab.

Despite previous research on the SIRE effect having varied widely with regard to its application, the fundamental underlying mechanisms are still in need of clarification. There are currently two major proposed theories that attempt to explain the SIRE effect: Christman et al.’s theory of interhemispheric interaction [5], and Lyle et al.’s theory of top-down attentional control [9]. Because the purpose of the current study was to conceptually replicate and extend existing work before moving on to further test these leading theories, we begin by examining the background rationale and literature supporting each account.

### Interhemispheric interaction

Christman et al. (2003) argued in the very first bilateral eye movement article that their observed memory benefit was due to increased interhemispheric interaction [5]. They based this hypothesis on their own previous work on handedness, which suggested that left-handed individuals have naturally greater interhemispheric interaction due to their larger corpus callosa, and therefore naturally perform better on episodic memory tests [7,20]. Christman et al. (2003) broadly based their interhemispheric interaction hypothesis of SIRE around the *Hemispheric Encoding and Retrieval Asymmetry* (HERA) [21,22] and *Cortical Asymmetry of Reflective Activity* (CARA) [23] hypotheses. While the HERA model suggests that encoding is mostly left-lateralized and retrieval is right-lateralized, the CARA model alters this slightly to propose that retrieval processes occur either primarily in the right hemisphere or in both hemispheres, depending on the task [23].

Following the logic of the HERA and CARA models, the interhemispheric interaction hypothesis argues that eye movements made before retrieval prime the hemispheres for interhemispheric activity so that tasks taking advantage of bi-hemispheric communication should receive a performance benefit [5,10]. According to this hypothesis (and the CARA hypothesis broadly), tasks with more ‘reflective activity’ (i.e., “when retrieval of additional information or more detailed evaluations of activated information are required during episodic remembering” [23, p. 3513]) will lead to the use of both hemispheres at retrieval [23,24], thus benefiting from an interhemispheric interaction priming effect. This proposal has been supported by work from Brunyé et al. [10], which showed a memory benefit following bilateral eye movements when participants performed a YES/NO recognition test, but not when they performed a two-alternative forced choice recognition test (2AFC) [10]. Their argument was that the YES/NO test makes use of more reflective activity than does the 2AFC test, and for that reason showed a performance benefit following bilateral eye movements [10].

Propper and Christman (2008) also supported the interhemispheric interaction hypothesis, in their case by connecting it to REM sleep processes [25]. It was suggested that because the majority of eye movements made during REM sleep cycles are horizontal [26], and that there is an increase in interhemispheric coherence during REM sleep [20], the SIRE effect mimics the same processes found in REM sleep, thereby resulting in increased interhemispheric interaction [26].

Importantly, the interhemispheric interaction hypothesis predicts that only bilateral eye movements (meaning, only *horizontal* movements) should result in a SIRE benefit. It has been shown that unilateral saccades activate the contralateral hemisphere [27]. In the context of SIRE, this would suggest that vertical eye movements should not produce a SIRE effect because they do not produce the same bilateral activation in frontal eye fields as is produced by horizontal eye movements [22,28]. In summary, the interhemispheric interaction hypothesis predicts that only horizontal eye movements should result in a performance benefit due to the inherent alternating activation of hemispheres. Thus, to test this theory, the current experiments measured memory performance following horizontal eye movements compared to both vertical and no eye movements.

### Top-down attentional control

A competing account soon emerged for the observed memory benefit following bilateral eye movements—that the boost resulted from top-down attentional control processes. Work by Lyle et al. was the first to propose that the SIRE effect could be due to the influence of eye movements on attention [29]. They suggested that since bilateral activation of frontal eye fields occurs even with vertical eye movements [30,31], this means that both types of eye movements should lead to a SIRE effect [29]. Indeed, others have reported that eye movements in any direction lead to frontal eye field and intraparietal sulcus activation, which themselves have been implicated broadly in attention processes [32,33]. Because past work has shown that frontal eye fields are so intertwined with other brain regions within the same attention network (e.g., the central sulcus, cingulate sulcus, medial frontal gyrus, and intraparietal sulcus) [32], Lyle et al. theorized that frontal eye field activity can lead to a cascading effect of activation that ultimately results in broad activation of both hemispheres [29].

As already mentioned, Lyle and Martin (2010) coined the term “SIRE effect” [9]. In the same article, they also refined the top-down attentional control hypothesis to further emphasize an attentional component as opposed to interhemispheric interaction. Their updated account suggested that engaging in bilateral eye movements requires top-down attentional processing to successfully perform repetitive purposeful eye movements, thus preparing the participant to make fewer errors, for instance, on subsequent tasks that require higher levels of attentional control [9]. Further, they proposed that eye movements are tied to activation in frontal eye fields and the intraparietal sulcus, which are both in turn part of a frontoparietal attention-control network [32]. Since the aforementioned attention network has been implicated in top-down allocation of attention during memory retrieval, eye movements in any orientation should theoretically prime this network. Priming of this network then ultimately aids top-down attention processes—and therefore memory performance—on a retrieval test [9].

After finding evidence of a SIRE effect following *vertical* eye movements [see also 29], Edlin and Lyle (2013) sought to more directly test their top-down attention control theory [11]. To do this, they employed a typical eye movement paradigm as used by others in the SIRE literature but instead employed a task designed to measure executive attention. Their results indicated that participants who made bilateral eye movements, relative to controls who made no eye movements, experienced greater attentional control as measured by faster reaction times on the ANT-R [11].

In 2015, Lyle and Edlin evaluated this attention idea again, opting to employ both horizontal and vertical eye movement conditions, along with a return to more common memory retrieval tests (namely, recall and recognition) instead of the attention test used previously [28]. Critically, some items on the recall test were made more difficult than others by first having participants practise recall from certain studied semantic categories but not from others. They then administered a final recall test, with the prediction that items from non-practised categories would be more difficult to retrieve. Lyle and Edlin performed this experimental manipulation because it has been shown in the literature that top-down attentional processes play a key role in memory tests, especially when performance is low and cognitive effort is high [34]. Results aligned with their predictions: The SIRE benefit was found when memory tasks were harder and therefore required greater top-down attentional control [28]. Crucially, they also found a memory performance benefit following vertical eye movements (for the second time), which is predicted only by their top-down attentional control account.

In summary, the top-down attentional control hypothesis suggests that purposeful eye movements (horizontal or vertical) lead to the priming of an executive function attention network that aids the allocation of top-down attentional control at retrieval, ultimately manifesting in a memory performance boost. Our experiments, much like for the previous theory discussed, compared memory performance following each of three critical eye movement conditions (horizontal, vertical, and centered) in an attempt to provide evidence in support of or in opposition to the top-down attentional control account.

### Neuroimaging evidence

Four studies to date have used neuroimaging methods (all electroencephalogram; EEG) to directly assess the interhemispheric interaction hypothesis as an account for benefits following bilateral eye movements. The first, conducted by Propper and colleagues (2007), employed EEG following bilateral eye movements to show that, relative to controls (who made no eye movements), there was decreased gamma band coherence between the two hemispheres in the bilateral eye movement condition [35]. That is, the authors actually observed *decreased* interhemispheric coherence (contrary to the increased interhemispheric interaction that Christman et al., 2003 suggested). They nevertheless argued that *any* changes in neural activity induced by eye movements that resulted in a boost in memory performance still broadly supported Christman and colleagues’ (2003) idea that changes in interhemispheric communication are key.

In the second relevant neuroimaging study, Samara and colleagues sought to test the interhemispheric interaction hypothesis using EEG [17]. Contrary to Propper and colleagues’ article, Samara et al. found *no* evidence for consistent alterations in interhemispheric interaction following bilateral eye movements across six frequency bands, including gamma [17]. It should also be noted that Samara et al. did not find a significant behavioral SIRE effect for neutral valence words, although they did find one for emotional words [17]. Ultimately, though, no difference was found in bilateral EEG coherence for either stimulus valence condition [17]. Thus, their results were entirely in contrast to those of Propper et al. [35] as well as being inconsistent with the broader interhemispheric interaction hypothesis.

Finally, the most recent two EEG studies that have investigated the effects of bilateral eye movements were performed by Fleck and colleagues. In the first, the authors found reduced posterior delta coherence in the control (center-eye movement) group relative to a bilateral eye movement group, suggesting that performing eye movements led to sustained executive attention [36]. In the second article, Fleck and his collaborators failed to find any behavioral differences between groups on the *revised attention network test* (ANT-R) following bilateral eye movements [37]. The authors did, however, find differences in N100 and P200 event-related potentials (ERPs), which due to these ERPs’ connection with selective attention [38,39], results were taken to indicate that less selective attention is needed on the ANT-R following eye movements [37]. Taken together, these two studies from Fleck and colleagues posit that participants experience a broad benefit for multiple aspects of attention following bilateral eye movements.

### Handedness considerations

In addition to eye movement orientation, the SIRE effect has also been reported to be rather sensitive to participant handedness. In fact, Christman and colleagues’ (2003) original article based their hypothesis concerning interhemispheric interaction on prior research from their own laboratory that suggested a similar neural substrate and episodic memory enhancement for participants with positive familial sinistrality (i.e., having left-handed relatives). They argued that this was likely due to left-handers’ naturally larger corpus callosa, which allows for greater interhemispheric communication [7; also see 20]. Their idea was that right-handers’ naturally smaller corpus callosa could benefit from an increase in interhemispheric communication that comes as a result of bilateral eye movements, whereas left-handed and mixed-handed individuals (with naturally larger corpus callosa) are already at such a high level of baseline interhemispheric communication that the eye movements provide no additional benefit [10]. Because Christman et al. [5] specifically selected their participants to be strongly-right handed and made the aforementioned case for handedness considerations, most research in this domain has followed suit and similarly only tested right-handed participants [e.g., 10].

A brief report by Lyle and colleagues demonstrated—in contrast to the enhancement seen for strongly right-handed individuals—a significant *reduction* in memory performance following bilateral eye movements for non-strongly right-handed individuals [29]. This is odd, though, because neither theory of the SIRE effect would have predicted memory attenuation for the latter group.

It is worth noting that Lyle and Edlin’s (2015) top-down attentional control theory did not consider right- versus left-handedness; instead, they stated that consistent handedness (often considered to be a score of 0.80 or greater on the Edinburgh Handedness Index) is the truly important factor [28]. They argued that only consistent handers seem to benefit from bilateral eye movements, but unfortunately they did not provide a solid prediction as to why this handedness inconsistency occurs [28]. Similar to the Christman et al. (2003) idea, Lyle and Edlin speculated that inconsistent-handers might have larger corpus callosa than consistent-handers, seemingly following along the lines of an interhemispheric interaction account for SIRE [28]. That is, consistent-handers with smaller corpus callosa theoretically have a lower baseline level of interhemispheric interaction and can therefore benefit from bilateral eye movements. Indeed, a recent review article summarizing handedness suggested ultimately that consistency may be a better predictor of interhemispheric interaction and right hemisphere processes than traditional left- or right-handedness distinctions [40]. Within the SIRE literature more specifically, Lyle and Edlin sum up perfectly that “the effect of saccade execution on inconsistent [handed] individuals is highly variable” [28, p191]. A potential explanation for these findings tracks well with an earlier presented argument from Brunye et al. [10]: inconsistent-handers have naturally larger corpus callosa and therefore might be at a ‘ceiling’ of interhemispheric interaction making them unable to benefit from bilateral eye movements.

In summary, while Christman et al.’s (2003) interhemispheric interaction account predicts that only right-handers (or in later literature, consistent-handers) should benefit from SIRE, Lyle and Edlin’s (2015) top-down attention account makes no differential prediction.

### Implications for eye movement desensitization and reprocessing (EMDR) therapy

The astute reader will have noticed that the SIRE effect literature employs a paradigm that is very similar to the technique used in *Eye Movement Desensitization and Reprocessing* (EMDR) therapy. When EMDR therapy is used to treat *Posttraumatic Stress Disorder* (PTSD)—the most common use for this type of therapy—the patient is typically instructed to make repetitive bilateral eye movements while recalling their traumatic experience [41; see 42 for a review]. This is one of the most critical factors warranting further exploration of the SIRE effect: Related therapeutic practices are basing their patient treatment on the efficacy of bilateral eye movements as a memory enhancement tool.

A common problem with implementing therapy techniques for those that suffer from PTSD is that patients’ dissociative amnesia of their traumatic memories makes it difficult to recall and work through their trauma in a therapeutic environment [see 43 for a review]. Christman et al. [5] suggested that the bilateral eye movements made during EMDR therapy play a similar role to the eye movements made in SIRE studies. That is, the role of eye movements in EMDR is to promote eased retrieval of episodic memories, and therefore to allow for therapeutic guidance to take place more effectively [see 25 for a review].

In addition to the reported episodic memory benefits following bilateral eye movements, there is some evidence that these same eye movements can also reduce an individual’s emotionality. If this were indeed the case, it would imply that SIRE is even more applicable for PTSD-related therapeutic interventions: Eye movements could not only allow patients to remember their repressed traumatic memories better, but they could also calm the patient’s emotional state, allowing for eased therapeutic guidance to take place. Christman and Propper (2008) reported a study in which horizontal eye movements were associated with a significant neutralization of mood among participants, such that previously happy and previously sad individuals became respectively less happy and less sad following bilateral eye movements. Bartels and colleagues (2018) reported that bilateral eye movements even caused a reduction in sexual fantasy vividness, arousability, and emotionality [44].

In relation to EMDR more specifically, Stickgold has argued that bilateral eye movements mimic *rapid eye movement* (REM) phase sleep processes [45]. Much like in REM sleep, it has been proposed that repetitive eye movements allow for enhanced cortical integration of memories, thus making the memories less dependent on the hippocampus (and, by extension, on the amygdala), ultimately reducing the emotionality of the memories. Although research on the emotional neutralization effects of SIRE is rather limited, the commonality of bilateral eye movement manipulation between SIRE and EMDR is obvious. However, mixed results and interpretations within both the EMDR [e.g., 46] and SIRE [e.g., 47,48] literatures have resulted in a rather controversial discussion surrounding the efficacy of both effects. Thus, the validity of both phenomena should be interpreted with caution.

Due to both the theoretical and real-world implications of SIRE for psychological research and therapy applications, the effect has not surprisingly intrigued investigators. First and foremost, therefore, we wanted to better understand the underlying mechanisms that may be at work causing a boost in memory performance. Believing that it is always wise to start with replication of a basic phenomenon before undertaking further research, we began with a conceptual replication of the SIRE effect in our laboratory.

## Experiments

### Experiment 1

The aim of Experiment 1 was to conceptually replicate the basic SIRE effect in our own laboratory before moving on to investigate the potential mechanisms. Because this was a conceptual replication, we tried to closely extend the original bilateral eye movement study by Christman et al. (2003). However, in contrast to the bulk of the bilateral eye movement literature, we opted to use a within-subject design to increase statistical power; there seemed to be no theoretical basis for design choice to alter the effect. In fact, the SIRE effect has already been reported when using a within-subject design [10,17].

In this first experiment, we asked participants to learn and remember a series of visually presented words, then to undergo a short unrelated filler task, followed by the critical eye movement task, and finally by an old/new recognition test.

#### Method

*Materials*. *Word lists*. Word lists of concrete nouns were derived from the *International Picture Naming Project* (IPNP) database [49]. In each list, average word length was 5.76 characters (*SD* = 1.75) with 1.69 syllables (*SD* = 0.71), and frequency was average (2.85; log-transformed CELEX; *SD* = 1.29). Of the created word lists, one would be studied at encoding for each eye movement condition, while another would serve as lures on the subsequent old/new recognition test. List assignments were fully counterbalanced between eye movement condition blocks, as was their assignment as to-be-remembered or as lure stimuli.

*Filler task tones.* Between study and test, a series of tones was presented as a one-minute test-delay filler task, which served the dual purpose of allowing for the upcoming memory test to assess long-term memory, as well as to guard against possible ceiling effects. Participants were asked to press 1, 2, or 3, on the keyboard to identify whether each tone was low (372 Hz), medium (498 Hz), or high (624 Hz), respectively, based on examples provided during the instructions and practice phase. Tones were presented approximately every second.

**Fig 1:**
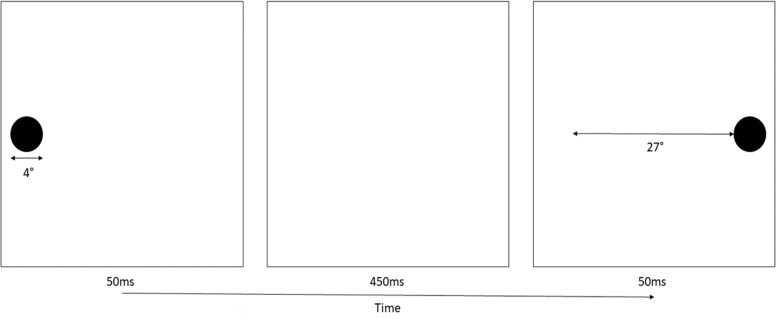
Eye movement phase timing and stimulus size.

*Eye movement stimuli.* In the horizontal eye movement task, participants were asked to view and visually track a single solid black dot extending approximately 4 degrees of visual angle in diameter, which sequentially appeared in left and right vertically-centered positions on a computer monitor. There was a blank space of approximately 27 degrees of visual angle between the inside edges of the two circle stimuli (see Fig 1).

The vertical eye movement task was very similar except that the dot stimuli now appeared at the top and bottom of the screen along horizontally-centered positions. All visual angle dimensions and stimulus timings were identical in the horizontal and vertical eye movement tasks. The centered (no eye movement) task used the same black dot stimulus and timing as in the eye movement tasks, but in this case the stimulus flashed repeatedly at the center of the screen. A chin rest was used throughout the experiment to ensure consistent visual angles across all participants. Participants’ heads rested roughly 46 cm from the screen throughout the experiment.

*Waterloo handedness questionnaire.* The Waterloo Handedness Questionnaire (36-item version) [50] was used to determine the extent of participant handedness on a continuum from left to right. Similar to the Edinburgh Handedness Index (EHI) commonly used in the bilateral eye movement literature [51], scores on the Waterloo Handedness Questionnaire can range from -1 (strongly left-handed) to +1 (strongly right-handed), with a score of 0 representing ambidextrous.

#### Procedure

Following informed consent, participants completed the *Waterloo Handedness Questionnaire* (WHQ), and were then seated in front of a 24-inch monitor connected to a Windows computer running *E-studio 3*.*0* [52]. The experimenter then asked the participants to position themselves comfortably in the table-mounted chin rest. The height of the chin rest was adjusted for participant comfort, but was also positioned such that the participant reported being able to stare straight ahead, looking at the center of the screen. The use of a chin rest in our study is not borrowed from any bilateral eye movement literature, but was employed to ensure consistent visual angle across both horizontal and vertical eye movement trials. The experimenter then read all instructions aloud, and participants were offered several opportunities to ask for clarification regarding the experimental procedures both before and after a practice block.

Participants completed one practice block, followed by three counterbalanced experimental blocks. During the practice block, participants completed an encoding phase, a tone-classification filler task, a small sample of each eye movement condition (one after another), and then a short old/new recognition test. Each experimental block was similar to the practice block, with the key differences being longer instances of encoding (2.25 minutes), filler task (one minute), and retrieval phases (one minute), as well as eye movement condition now being blocked (horizontal, vertical, or no eye movements, each for 30 s).

During the encoding phase, 30 words were presented one at a time. A single encoding trial consisted of a fixation cross presented at the center of the screen for one second, followed by presentation of the to-be-remembered word for three seconds, and finished with a blank screen for 500 ms. Matching the methods outlined in Christman et al. (2003), words were presented in 28 pt upper-case Courier font [5].

During the post-study maintenance period, a tone-classification filler task was used to create a retention interval with the dual purposes of guarding against potential ceiling effects and ensuring use of long-term memory by minimizing any possible recency effect [e.g., 53]. During this task, participants were asked to use the keyboard to indicate whether each tone played through the computer speakers was of low, medium, or high pitch. Tones lasted roughly one second and, once a response key was pressed, the next tone played immediately. This continued for one minute. Within the SIRE effect literature, introducing a delay prior to test is common. For instance, Christman et al. [5] and Samara et al. [17] both used a 30-minute retention interval. Outside of the bilateral eye movement literature, this specific tone classification task has been used for several decades to absorb attention [54] and has been employed for the same purposes as our own in many recent studies [e.g., 55–58].

**Fig 2:**
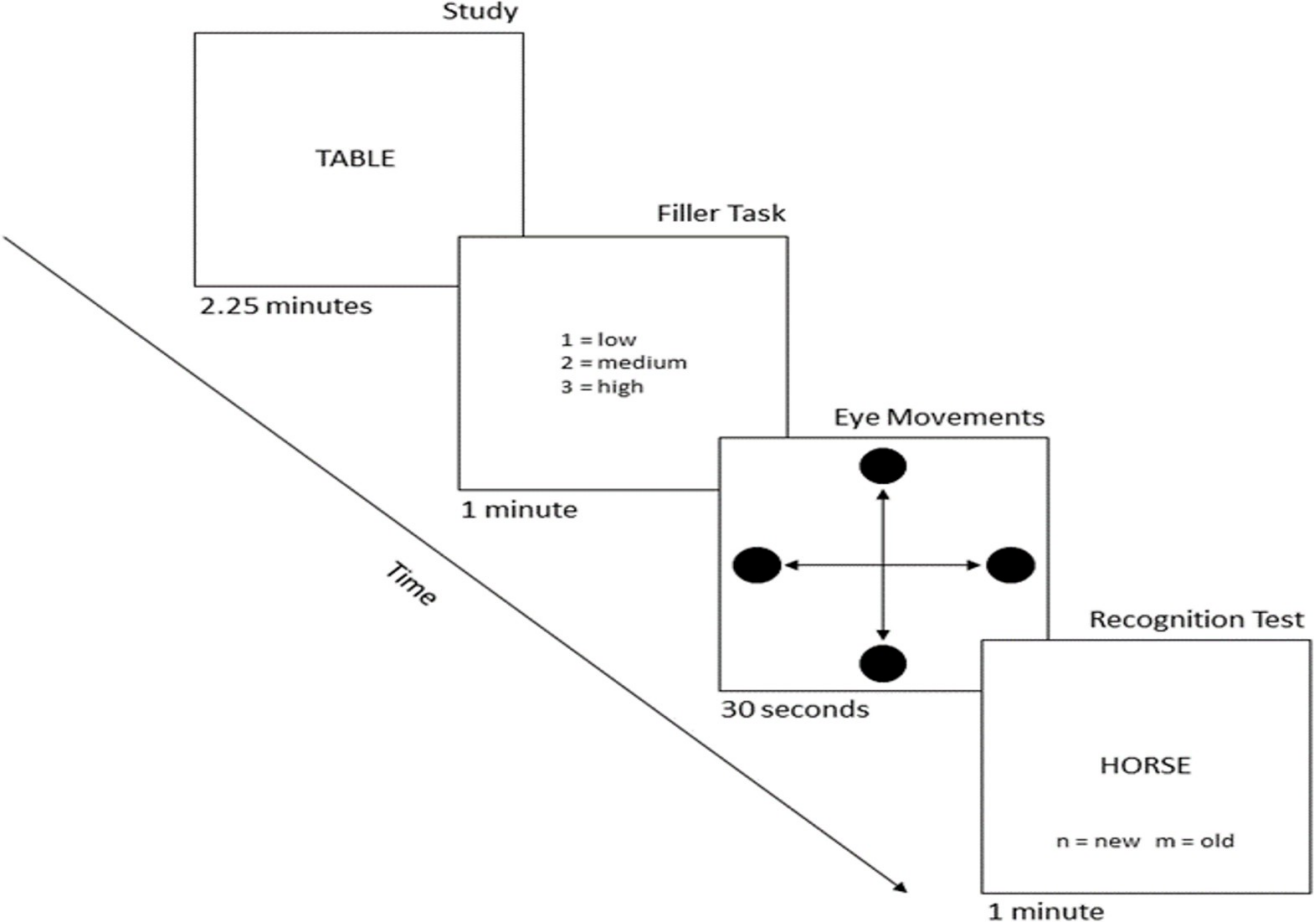
Overall study procedure used for each experimental block. In the critical manipulation, participants experienced one of three eye movement conditions (horizontal, vertical, or no eye movements), as depicted by the black dots with arrows to denote the expected direction of eye movement.

Following the filler task, participants underwent each of the three eye movement conditions, one per block (see Fig 2). These tasks were designed to closely extend previous work by Christman and colleagues, thus stimulus presentation and timing details followed their procedure [5]. In the horizontal eye movement block, participants watched a black dot flash in left and right positions on the monitor for a total of 30 s while following it with their eyes by making repetitive saccades. The vertical block was identical to this except that the black dots now appeared at the bottom and top of the screen. Finally, in the centered (no eye movement) condition, the black dot flashed at the center of the screen for 30 s and no saccades were made. The black dot stimuli were presented on the screen for 50 ms, with a 450 ms inter-stimulus interval before the next dot appeared, for an overall rate of roughly two presentations per second (and therefore two saccades in the vertical and horizontal conditions).

Immediately following the eye movement phase, participants were presented with an old/new recognition test. Thirty words were presented in random order, consisting of 15 randomly chosen target words and 15 randomly chosen lures. Only half of the 30 studied items were presented at test to reduce experiment run time. Participants responded ‘old’ or ‘new’ by pressing keys labeled as such (M or N, respectively on a QWERTY keyboard). Response time and accuracy for each trial were recorded using *E-Prime* Version 3.0 [52] software. Overall, each recognition test took approximately one minute for participants to complete.

Participants were given a five-minute break between experimental blocks to mitigate any possible carry-over effects. Without this break, there could be concern that any effects of eye movements (or lack thereof) on memory performance could persist into the next block. In the context of the two leading theories, this could take the form of persistent interhemispheric coherence or executive functioning attention boosts. As Brunye et al. noted in their SIRE study employing a within-subject design, transcranial magnetic stimulation (TMS) of the human cortex typically only lasts three to four minutes [59]. Thus, the logic is that even if bilateral eye movements produce effects with a duration and intensity similar to that of TMS, there should be at least a three-minute to four-minute break between blocks.

During the five-minute break, participants were informed that they could leave the chin rest and relax by taking a drink or using their mobile devices. After five minutes, a brief tone was played to notify participants that it was time to get back into the chin rest and resume the study. Once the participants had completed the last experimental block, they were informed of the completion of the study, thanked for their participation, and offered a detailed feedback letter.

All procedures and materials were approved by the Office of Research Ethics at the University of Waterloo (ORE #22799). Data and materials for our experiments are available on the *Open Science Framework* at https://osf.io/39uth/.

*Participants*. Within the original bilateral eye movement effect article from Christman et al. [5], an effect size of *d* = 0.495 was found for the critical comparison of horizontal eye movements to no eye movements. We therefore performed an *a priori* power analysis (matched-pairs, two-tailed t-tests with a *d* = 0.495) using G*Power software [60], which indicated a required sample size of 35 participants to achieve 80% statistical power. Thus, we aimed to collect 35 participants at minimum in each of our experiments.

In Experiment 1, 42 right-handed University of Waterloo undergraduates (30 female), ranging in age from 17 to 31 (*M* = 20.91, *SD* = 2.54), were recruited to participate for course credit. Participants had all self-reported to be right-hand dominant, to have normal or corrected-to-normal vision, and to have learned English before the age of nine. The Waterloo Handedness Questionnaire (WHQ) [50] indicated the average handedness score to be 0.65 (*SD* = 0.17, range = .32 to .99), indicating moderate right-handedness among participants.

#### Results

*Eye movements and memory sensitivity*. Before performing any statistical tests, we analyzed for univariate outliers in the data based on memory sensitivity (d prime; *d’*). By using a critical cut-off of three standard deviations above and below the mean on the memory sensitivity measure, we identified and subsequently excluded data from three participants that were univariate outliers, resulting in a remaining sample size of 39.

Within all of the experiments contained within this article, d-prime (*d’*) was used as a memory discriminability index, normalized criterion *c* (*c’*) as a measure of response bias, and Cohen’s *d* as a measure of effect size. Throughout this manuscript, in any case where a participant’s number of false alarms equalled zero or number of hits equalled one, we used a standard correction to these values before calculating *d’* scores [61]: False alarm rates were adjusted to 0.5/N where N is the number of lure trials; similarly, hits were adjusted to (N—0.5)/N where N is the number of target trials. In addition, for consistency, paired-samples Bayesian t-tests were used throughout our analyses. All of these t-tests were two-tailed and used a default Jeffreys-Zellner-Siow (JZS) prior for a medium effect size (Cauchy 0, 0.7071) [see 62–65]. This is the default uninformative prior used in *SPSS* [66], *JASP* [67], and the *BayesFactor* [63] package for *R* [68]. Bayesian analyses were employed in addition to traditional null hypothesis significance testing with the intention of offering the reader an alternative means of data interpretation, as well as the ability to quantify evidence in favour of a null effect.

**Fig 3:**
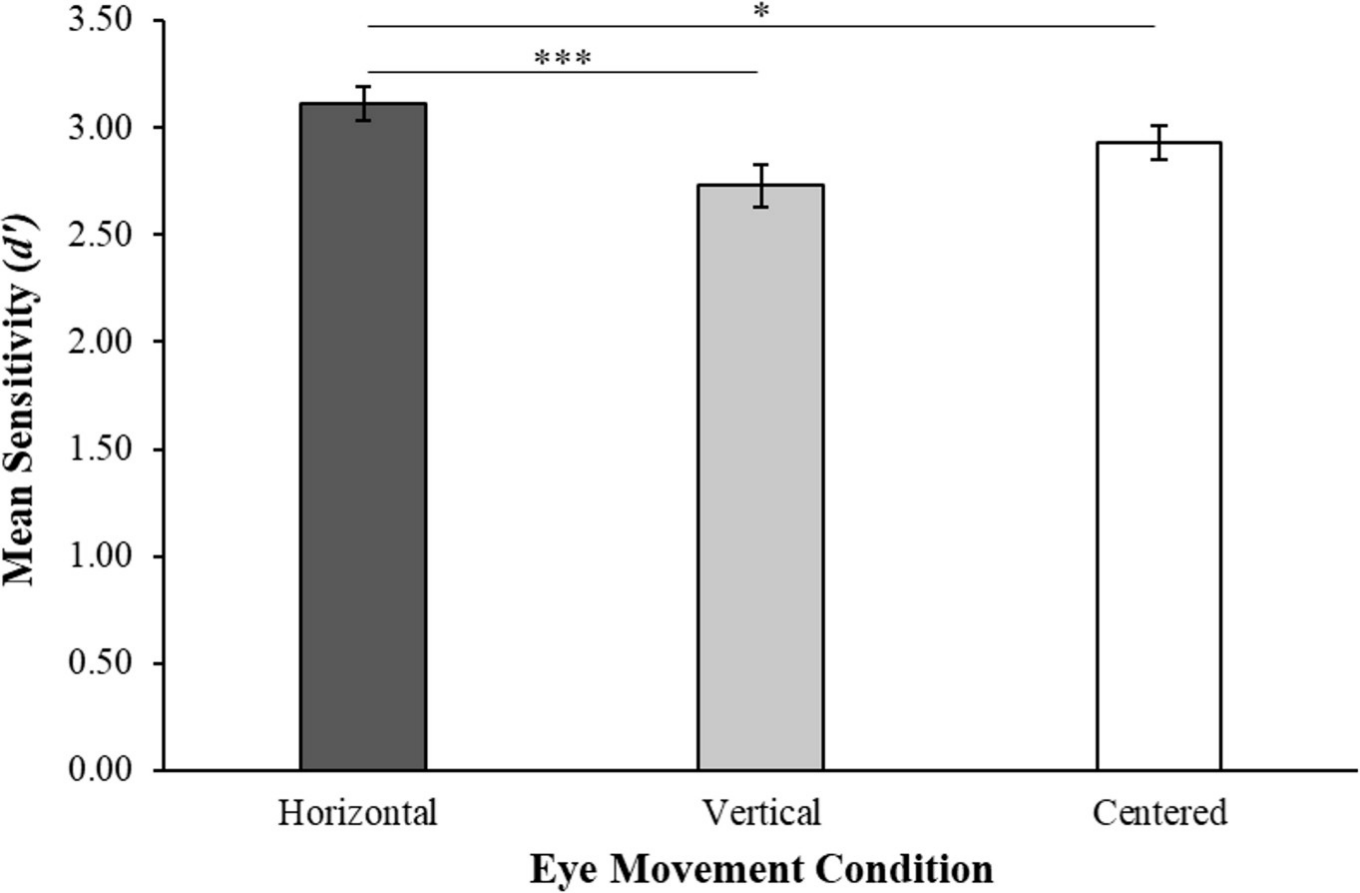
Experiment 1. Mean sensitivity (d prime) on the recognition test following each eye movement condition. Error bars represent ± 1 *SE*. Outlier data are not shown here. * = *p* < .05, *** = *p* < .001.

Results of the memory test, as measured by memory sensitivity (*d’*), revealed a significant main effect of eye movement condition, *F*(2, 66) = 7.28, *p* = .001, ηp^2^ = .175 (see Fig 3). Bayesian paired-samples t-tests revealed that memory sensitivity in the horizontal eye movement condition (*M* = 3.11, *SD* = 0.50) was significantly greater than in the centered condition (*M* = 2.93, *SD* = 0.49), *t*(38) = 2.27, *SE* = 0.08, *p* = .03, *d* = 0.36, *BF*_10_ = 1.40. The same statistical method also showed that memory sensitivity was significantly higher in the horizontal relative to the vertical (*M* = 2.73, *SD* = 0.61) eye movement condition, *t*(38) = 3.92, *SE* = 0.10, *p* < .001, *d* = 0.55, *BF*_10_ = 5.05, but that the vertical and centered conditions did not differ, *t*(38) = 1.88, *SE* = 0.11, *p* = .07, *d* = 0.23, *BF*_01_ = 1.53. Table 1 displays the means and standard deviations of hits, false alarms, memory sensitivity (*d’*), and response bias (*c’*) on the recognition tests.

**Table 1:** 

Eye Movement Condition	Hit Rate	False Alarm Rate	*d'*	*c'*
Horizontal	.88 (.12)	.08 (.09)	3.11 (0.50)	0.05 (0.12)
Vertical	.77 (.18)	.12 (.13)	2.73 (0.61)	0.13 (0.28)
Centered	.83 (.13)	.12 (.13)	2.93 (0.49)	0.06 (0.15)

Experiment 1 means (and standard deviations) for hit rate, false alarm rate, *d'*, and *c'*.

#### Discussion

In this experiment, horizontal eye movements led to superior memory relative to both vertical eye movements and a no eye movement control, the latter two of which did not differ from each other. The results of this experiment are broadly consistent with the findings reported by Christman et al. [5]. Our findings are also consistent with their interhemispheric interaction account for the SIRE effect—that horizontal eye movements will activate both hemispheres—due to the fact that only horizontal eye movements increased participants’ memory sensitivity. The results are not, however, consistent with the top-down attentional control account of Lyle and Martin, as their hypothesis would have predicted comparable memory performance following both horizontal and vertical eye movements [9]. Critically, one must recognize that Bayesian evidence for the horizontal versus no eye movement comparison was relatively weak (BF_10_ < 3) [69]. According to Jeffreys, a Bayes factor less than three indicates anecdotal evidence that “would be hardly worth mentioning” [69, p432]. Nevertheless, using classic null hypothesis significance testing standards, we did find a significant SIRE effect here. To our knowledge, this is the first experiment that has replicated (albeit weakly) the basic SIRE effect using a within-subject design in combination with neutral valence word-based stimuli.

### Experiment 2

As the Bayesian evidence for a SIRE effect was weak in Experiment 1, we sought to conduct a further conceptual replication. In Experiment 2, horizontal and vertical eye movement conditions were manipulated separately, and sample size was increased.

To achieve this, we employed the same methodology as in Experiment 1, which also in many ways followed that of Christman et al.’s original procedures [5]. The only deviations from Experiment 1’s protocol were the number of eye movement conditions experienced by a participant and the length of the recognition test. While in Experiment 1 each participant took part in all three eye movement conditions, in Experiment 2 participants were split into two groups. Group 1 experienced horizontal and centered eye movement conditions, within-subject; Group 2 experienced vertical and centered eye movement conditions, also within-subject. The purpose of this change was to split up the eye movement orientation types among participants to decrease any effect of interference or carry-over between eye movement conditions. That is, by splitting up vertical and horizontal eye movement blocks and comparing them each to the centered eye movement condition, we simplified the study to stand the best chance of observing a significant SIRE effect. Therefore, this was a mixed design in which the *critical* comparisons (active eye movement blocks to no eye movement blocks) were made within-subject. The recognition test was also doubled in length to prevent possible ceiling effects in memory performance.

#### Method

*Materials*. The materials used in Experiments 1 were also used here.

*Procedure*. The procedure was similar to that in Experiment 1 (including 5-minute breaks between blocks and use of a tone classification filler task), except that we adopted a mixed study design and doubled the length of the recognition test.

As mentioned above, eye movement conditions were separated in this study for the purpose of guarding against any possible carry-over or interference that might result from switching between eye movement conditions. Therefore, this experiment used a mixed design, with each participant randomly assigned to either the horizontal group (horizontal-centered; H-C; *n* = 50) or the vertical group (vertical-centered; V-C; *n* = 51), and eye movement condition being varied within-subject.

In addition, to guard against any potential ceiling effects, the recognition tests were doubled in length and included all 30 studied targets as well as 30 randomly chosen lures. Since the recognition test length was doubled, it now took participants approximately two minutes to complete each recognition test.

*Participants*. A total of 101 right-handed University of Waterloo undergraduates (79 female) were recruited to participate for course credit; they ranged in age from 17 to 32 (*M* = 19.31, *SD* = 2.39). Seven of these original participants’ data files had to be excluded due to technical errors and were subsequently replaced with data from new participants. The average WHQ handedness score of this sample was 0.67 (*SD* = 0.13, range = .25 to 1), again indicating moderate right-handedness. Participant selection criteria were identical to those of Experiment 1.

#### Results

*Eye movements and memory*: *Sensitivity*. We used the same statistical methodology as in Experiment 1. Three univariate outliers (± 3 *SD*) were detected and removed from this dataset, resulting in a final sample size of 49 participants in each of the two groups (total *N* = 98). Table 2 summarizes the means and standard deviations from the recognition tests.

**Table 2:** 

Group	Eye Movement Condition	Hit Rate	False Alarm Rate	*d'*	*c'*
H-C	Horizontal	.79 (.18)	.18 (.11)	2.01 (0.97)	0.07 (0.26)
	Centered	.79 (.15)	.18 (.15)	2.01 (1.04)	-0.03 (0.47)
V-C	Vertical	.75 (.16)	.18 (.14)	1.89 (1.03)	0.11 (0.38)
	Centered	.76 (.17)	.18 (.13)	1.89 (0.94)	0.10 (0.30)

Experiment 2 means (and standard deviations) for hit rate, false alarm rate, *d'*, and *c'*.

**Fig 4:**
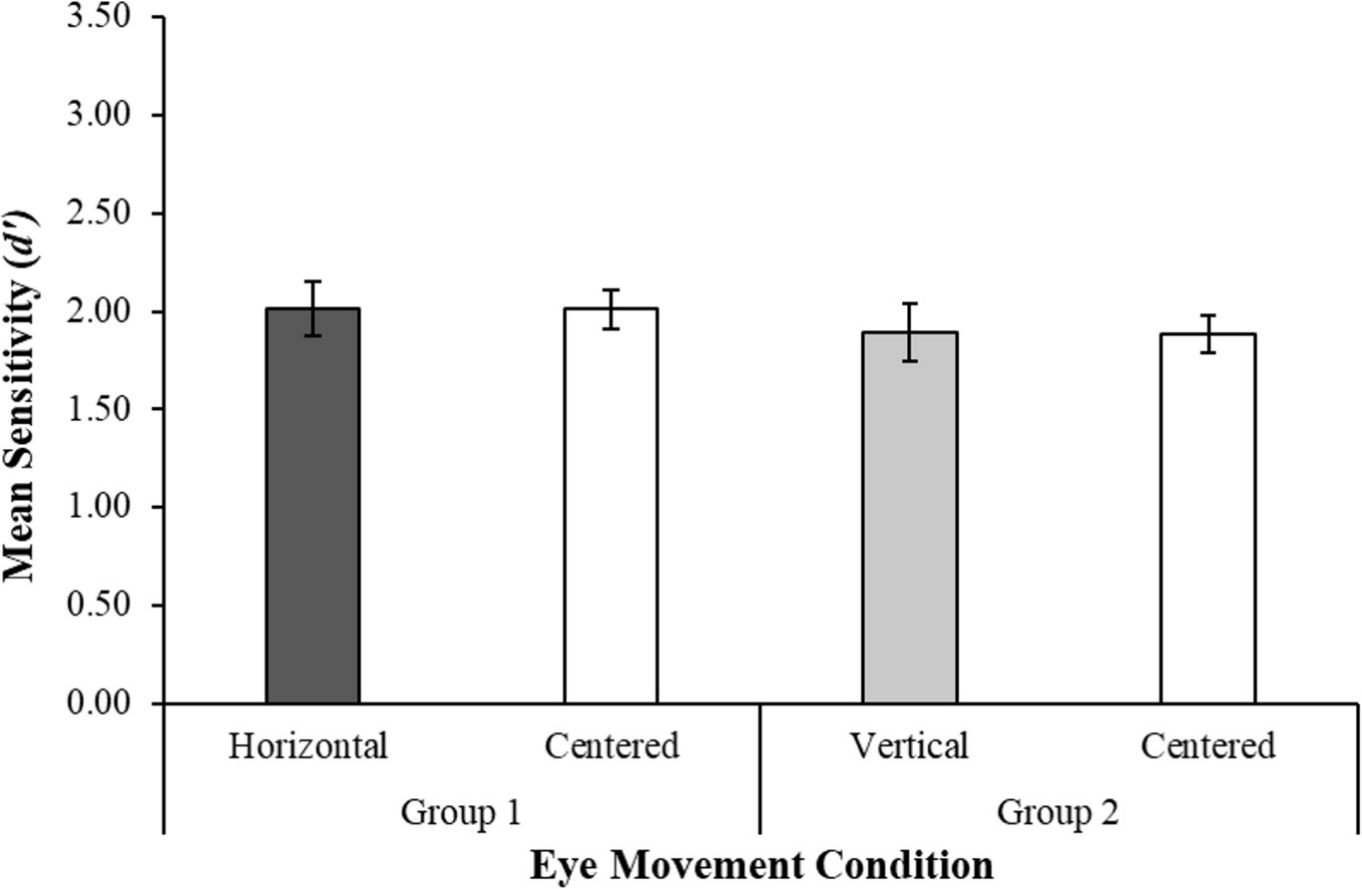
Experiment 2. Mean sensitivity (d prime) on the recognition test following each eye movement condition. Error bars represent ± 1 *SE*. Outlier data are not shown here.

A Bayesian paired-samples t-test in the horizontal group revealed that memory sensitivity was equivalent in the horizontal eye movement condition and the centered condition, *t*(48) = 0.13, *SE* = 0.13, *p* = .90, *d* = 0.02, *BF*_01_ = 8.87 (see Fig 4). Likewise, in the vertical group, memory sensitivity was equivalent in the vertical eye movement condition and the centered condition, *t*(48) = 0.21, *SE* = 0.10, *p* = .84, *d* = 0.03, *BF*_01_ = 8.76.

Unique to this experiment, an independent-samples Bayesian t-test was also conducted to compare the horizontal and vertical eye movement conditions between groups. There was no significant difference in memory sensitivity between the two eye movement conditions, *t*(96) = 0.71, *p* = .48, *d* = 0.15, *BF*_01_ = 5.09. Our aforementioned *a piori* power estimates were calculated assuming a paired-samples test, however, so it is possible that this statistical test was not sufficiently powered to detect an effect between-subjects.

*Eye movements and memory*: *Correlations with handedness*. Bayesian Pearson correlations were used to assess the influence of handedness on memory across our studies. Because we used the same measure of handedness across our experiments, we were able to collapse for a larger overall sample. Table 3 presents the results of these analyses.

**Table 3:** Associations between handedness and memory across all three experimental eye movement conditions.

Eye Movement Condition	Correlation with WHQ Score
Horizontal	*sr*(126) = .06, *p* = .53, *BF*_01_ = 11.77
Vertical	*r*(127) = -.11, *p* = .23, *BF*_01_ = 6.91
Centered	*r*(138) = -.01, *p* = .93, *BF*_01_ = 14.88

Bayesian Pearson correlations of Waterloo Handedness Questionnaire (WHQ) scores to memory sensitivity across the 2 main experiments and the supplemental experiment.

#### Discussion

Due to weak Bayesian evidence in Experiment 1, we sought to determine whether the effect was present in a modified design. We increased the sample size, and applied a within-subject methodology to increase statistical power even further (a greater chance of finding a true effect). We even went so far as to split up horizontal and vertical eye movement conditions, in case there were any carry-over effects of one saccade orientation on the other.

Despite all these features, Experiment 2 failed to find evidence for a SIRE effect, with Bayes factors indicating substantial evidence for a null (3 < BF_01_ < 10) [69]. A failure to find the SIRE effect here, despite our increased power, validated methodology from Experiment 1, and conceptual replication of previously published methods, suggests that the SIRE effect as reported in the literature is fragile at best. This claim is considered further in the General Discussion.

*Participant handedness considerations*. There has been one difference in our participant samples relative to other work: handedness. It has been argued by some [e.g., 29] that participant handedness can have a moderating effect on any subsequent memory boost that bilateral eye movements provide, such that only strongly right-handed (or consistent-handed) individuals should experience a memory benefit (see our introduction for more on this). Furthermore, Lyle et al. even showed a small but significant decrease in memory performance following bilateral eye movement in left-handed individuals [29].

Normally in the SIRE literature, participants are split *a priori* into left-, right-, consistent-handed, or inconsistent-handed groups to assess handedness effects. Typically, a score of 80 on the Edinburgh Handedness Inventory (EHI) is used to indicate whether participants are consistently handed or not. Instead of following this method and assigning participants to groups based on handedness scores, we opted to assess the role of handedness on memory across our studies using Bayesian Pearson correlations. The idea is that because all of our participants were right-handed, then as handedness scores rise, so should memory performance following bilateral eye movements (i.e., a positive correlation between handedness consistency and size of the SIRE effect). As is clear from Table 3, there was no significant correlation between handedness scores and memory performance within any eye movement condition. In fact, the Bayes factors demonstrate moderate to strong evidence for a null effect.

It is also worth noting that whereas some studies in the SIRE literature demonstrate an effect of handedness on SIRE-related memory performance outcomes [e.g., 29], others do not include any metric of handedness information [e.g., 35,44]. An article by Samara et al. did in fact report handedness of participants but found no evidence for the basic SIRE effect in their strongly right-handed participants when testing neutral valence words (contrary to the prediction of both leading hypotheses), but did find evidence for a SIRE effect when they used emotionally valenced words [17]. Additionally, Edlin and Lyle also reported non-significant SIRE by handedness interactions [11]. Finally and most importantly, Matzke et al. employed a criterion of only allowing for strongly right-handed participants in their study (as indicated by the traditional 80 split on the EHI) and failed to find the SIRE effect [16]. Thus, our article joins other work within the related literature suggesting that handedness may not be as important a factor to the SIRE effect as some have previously theorized.

## General discussion

Overall, the present results do not support the claim that eye movements prior to retrieval consistently lead to improved memory performance. Across two experiments, we found the effects of bilateral eye movements on memory performance to be very weak or simply absent, suggesting that the effect could be especially sensitive to experimental design factors. Regardless of the direction of eye movements, we observed no consistent change in memory performance.

The inconsistent replicability of the SIRE effect in our study, together with the findings from other studies failing to observe a significant effect [16–17] suggests a reconsideration of its reliability and usefulness as a clinical tool. Perhaps it is the case that bilateral eye movements are beneficial only when participants are of a particular handedness demographic. Perhaps it only shows up when between-subjects designs are used. Perhaps it only works in some laboratories because of slightly different manipulations. If the SIRE effect is indeed this sensitive to experimental methodology, its reliability as a scientific effect, and its utility in clinical applications, should be questioned. At the very least, its boundary conditions should be made clear. It is important that cognitive effects observed in the laboratory be reliable and easily replicable—and ideally generalizable—before they can be applied clinically, as in EMDR therapy wherein real-world mental health outcomes are at stake. Although the current experiments did not directly test EMDR, the cognitive mechanism proposed as following bilateral eye movements is strikingly similar in the two situations. Therefore, the present results provide a cautionary tale regarding the reliability of eye movements as a tool to change aspects of cognition, even when employed in closely related therapeutic methods.

Given the appeal of an emergent body of work arguing for the benefit of bilateral eye movements on memory performance, consistent replication is required. Although our study was not a registered report with the goal of directly replicating previous work, the methodology that we employed did quite closely follow that used in previously published work [e.g., 5]. Importantly, our results do not stand alone in at least sometimes failing to observe a SIRE effect; others have also failed to replicate the effect [16–17]. Still, it is important to consider methodological differences and limitations that existed within our own experiments that may have influenced our results.

### Between- vs within-subject designs

One of the largest differences between our work and that of the broader SIRE literature is the type of study design used. Whereas the literature has largely used between-subjects designs, we used a within-subject design. Our design choice was made for the purpose of increased statistical power, given no reason to anticipate that this would alter the effect. Still, when using a within-subject design, one must be wary of carry-over effects. To our knowledge, only two other SIRE articles used within-subject designs. Brunyé et al. did so and also employed a 10-minute delay between blocks to mitigate carry-over effects [10]. Although they did find a SIRE effect in their study, they did not mention any carry-over effects in their results. Samara et al. also used a within-subject design (with a one-week delay between sessions) and failed to find the SIRE effect for neutral words (but curiously did find it for emotionally valenced words) [17]. Similar to the work by Brunyé and colleagues, Samara and colleagues mentioned counterbalancing, but no order effects were reported.

Within the present experiments, we used a five-minute delay between blocks. We did analyze for order effects but found nothing reliable. Further, other memory performance enhancing phenomena, such as the production [70], generation [71], and drawing [57] effects, have been demonstrated to be most pronounced when using within-subject designs. The broader point both from our experiments and from the related literature remains: Using a within-subject relative to a between-subjects design should not moderate the SIRE effect in any detrimental way and, due to reduced overall variance, should even provide a better chance of finding the effect.

### Looking forward

As scientists in our field are undoubtedly aware, there is an ongoing ‘replication crisis’ [e.g., 72]. Thus, it seems beneficial to discuss potential options for bettering ourselves as a broader scientific community moving forward.

Matzke et al.’s attempted replication serves as a solid foundation upon which to build [16]. Their choice of conducting a pre-registered adversarial collaboration is undoubtedly a step in the right direction, but more needs to be done. Ideally, a multisite replication effort (perhaps using the Psychology Science Accelerator) could be formed involving leading SIRE effect researchers to provide a quality, pre-registered replication attempt with high statistical power and agreed-upon methods. We believe that this form of collaborative replication would be the best way to evaluate the validity of the SIRE effect.

Further, repeated replication attempts of the SIRE effect also inform related work in the field of *Eye Movement Desensitization and Reprocessing* (EMDR) therapy. As was discussed earlier, the rationale and methodology of SIRE and EMDR therapy seem clearly linked—both involve repetitive bilateral eye movements to improve memory retrieval [5,41]. Discussion surrounding the efficacy of EMDR therapy is arguably even more contentious than that of the SIRE effect [e.g., 46]. More importantly, the worthiness of EMDR therapy has direct mental health and financial implications for vulnerable populations, such as those with PTSD. Why have people subjected to something that has little to no empirical base of evidence? Thus, a secondary benefit of further replicating the SIRE effect is that it provides evidence in regard to mechanisms that may also underlie EMDR therapy.

Ultimately, further replication in this literature will only serve to better our field. For instance, documenting boundary conditions on the effect may lead to even more interesting theoretical contributions. Often, a failure to replicate can indicate a nuance in methodology or participant demographics that had not yet been considered. Such findings could serve to refine and re-evaluate existing theories.

## Supporting information

S1 Fig Supplemental experiment. Mean sensitivity (d prime) on the recognition test following each eye movement condition. Error bars represent ± 1 *SE*.
(TIFF)

S1 File Supplementary experiment [5, 7, 67, 73]. (DOCX)
