# Supplementary figures and images for: Re-evaluating whether bilateral eye movements influence memory retrieval
Source: PLoS One. 2020 Jan 27;15(1):e0227790. doi: 10.1371/journal.pone.0227790 (PMC6984731; doi:10.1371/journal.pone.0227790)

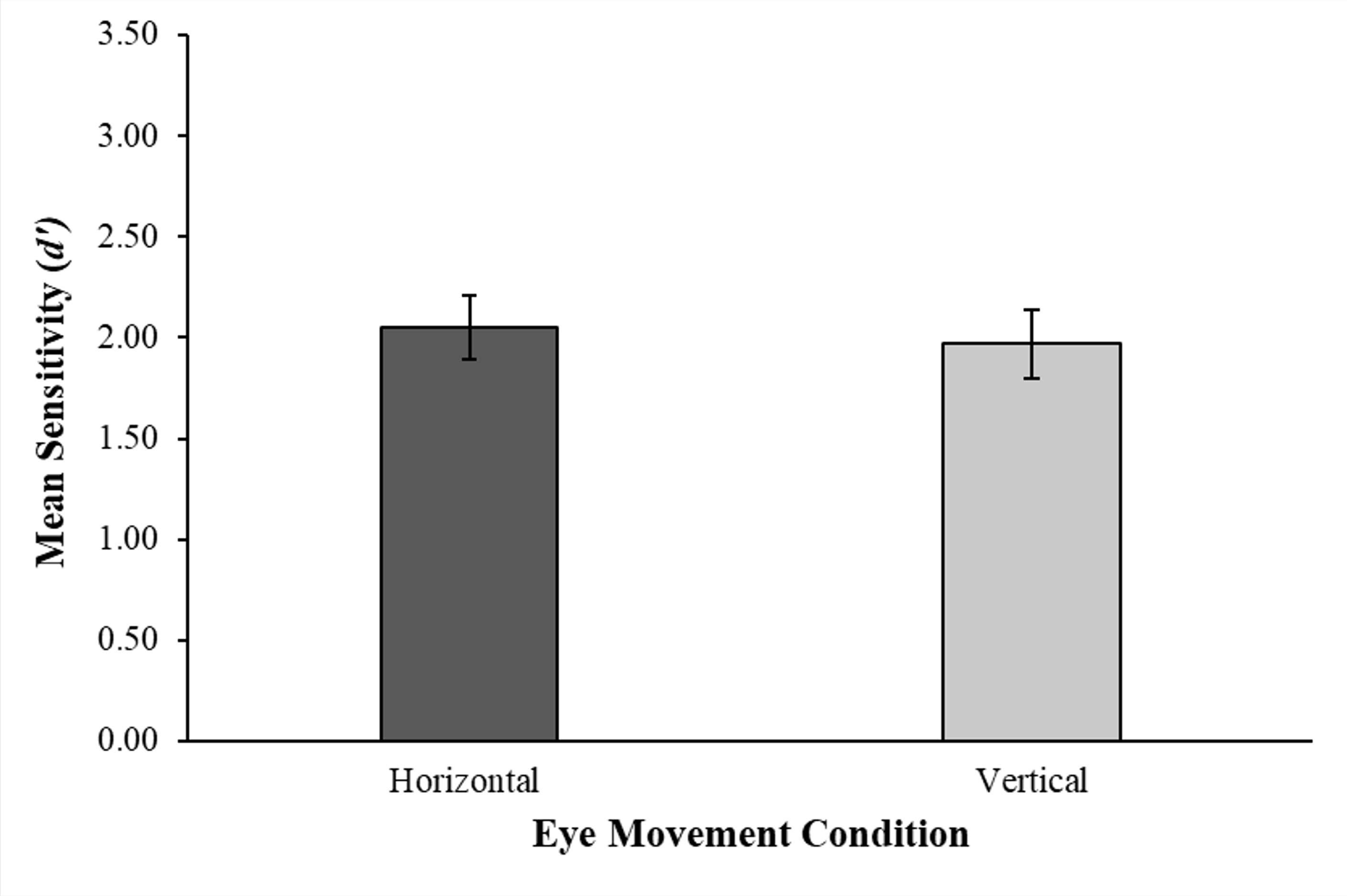

Supplement: S1 Fig — Mean sensitivity (d prime) on the recognition test following each eye movement condition. Error bars represent ± 1 SE. (TIFF) [file pone.0227790.s001.tiff]
