# Supplementary material for: Re-evaluating whether bilateral eye movements influence memory retrieval
Source: PLoS One. 2020 Jan 27;15(1):e0227790. doi: 10.1371/journal.pone.0227790 (PMC6984731; doi:10.1371/journal.pone.0227790)
Supplement: S1 File — (DOCX) [file pone.0227790.s002.DOCX]

**Supplementary experiment**

Experiment 1 showed that conceptual replication of the SIRE effect was possible using the word list, test delay time, and within-subject design that was outlined in our Method. Given this, we sought to conceptually replicate again, this time using auditory stimuli. We chose to use auditory stimuli because this would allow for increased flexibility in future experiments in which we aimed to investigate the influence of eye movements performed *during* the encoding phase. For instance, we imagined a study design in which eye movements are made in response to visual dot stimuli on the screen (as was the case in Experiment 1), while simultaneously encoding auditorily presented words. Given this plan, the use of auditory stimuli with the SIRE effect required validation before we could address these pertinent questions.

**Method**

**Materials**

The materials used in Experiment 1 were also employed here.

**Procedures**

The procedure for the current experiment followed that of Experiment 1, save for three main differences: use of auditory stimuli, removal of the centered eye movement condition, and an (unintentional) reduction in saccade frequency.

During the encoding and retrieval phases, words were presented auditorily one at a time through computer speakers. A single encoding trial consisted of a fixation cross presented at the center of the screen for one second, followed by a three-second period during which a to-be-learned word was presented auditorily. The trial finished with a blank screen for 500 ms. Similarly, at the time of test, words were presented once auditorily and did not advance until a keypress indicating ‘old’ or ‘new’ was made.

The centered eye movement condition was removed to reduce the time and resources needed to run the experiment. We saw the horizontal-vertical comparison as of more theoretical interest, given that it equated factors such as eye movement stimulation and practising overt shifts of attention, leaving only the direction of eye movements as the critical factor driving any subsequent memory benefit.

Finally, saccade rate during the horizontal and vertical eye movement tasks was reduced by roughly half (one saccade per second). This was actually due to an experimenter error during programming. However, examination of the literature produced no theoretical reason that this would alter the effect. Obviously, there would likely be a difference between making one saccade during the task versus one hundred, but the difference here was not nearly as extreme—30 saccades versus 60 saccades.

**Participants**

A total of 39 right-handed University of Waterloo undergraduates (24 female), ranging in ages from 18 to 31 (*M* = 20.54, *SD* = 2.87), were recruited to participate for course credit. The participant criteria used in Experiment 1 were duplicated here. The average WHQ handedness score of this sample was 0.60 (*SD* = 0.16, range = -.06 to .94), again indicating moderate right-handedness among participants.

**Results**

**Eye movements and memory sensitivity**

In this experiment, we used the same statistical methodology as in Experiment 1. No statistical outliers were detected in this dataset. S1 File (S1 Table) summarizes the means and standard deviations of the memory scores from the recognition tests.

| **Supplementary Table 1** |  |  |  |  |
| --- | --- | --- | --- | --- |
| Eye Movement Condition | Hit Rate | False Alarm Rate | *d'* | *c'* |
| Horizontal | .81 (.14) | .18 (.17) | 2.05 (0.98) | -0.16 (-0.02) |
| Vertical | .82 (.12) | .21 (.19) | 1.97 (1.07) | 0.29 (0.28) |

Supplemental experiment. Means (and standard deviations) for hit rate, false alarm rate, *d'*, and *c'*.

A Bayesian paired-samples t-test revealed that memory sensitivity in the horizontal eye movement condition did not differ significantly from that in the vertical condition, *t*(38) = 0.57, *SE* = 0.14, *p* = .57, *d* = 0.07, *BF*_01_ = 6.83 (see S1 Fig).

**Discussion**

Since this experiment was run after the significant effect found in Experiment 1, the failure to replicate came as a surprise. Experiment 1 demonstrated that our changes in word lists, test delay, and use of within-subject design should all still result in a significant SIRE effect. Yet here, Bayesian evidence for a null effect was substantial (3 < BF_01_ < 10) [67].

Although this experiment did largely use the same methods as Experiment 1, there are two differences unique to this experiment that could have led to the observed null effect. First, eye movements made in this experiment were at a rate half that of Experiment 1. This resulted in one eye movement per second, rather than two as was previously used. To our knowledge, no work has been done in the related SIRE literature that would indicate any influence of this factor in determining memory performance. It is the case that most researchers opt to use Christman et al.’s original methodology, which outlines 30 s of eye movements at a rate of two per second [5]. Still, we have not found any research to suggest that number of saccades should matter: It remains an empirical question. If this does turn out to be a potent factor, this would place an important boundary condition on the SIRE effect.

Second, stimuli in the current experiment were presented auditorily at both encoding and retrieval. This presentation format was selected with the intention of replicating the effect using auditory stimuli before extending this method in subsequent experiments. Although the present failure to find the SIRE effect could be due to the use of auditory stimuli, this also does not seem likely. Parker and Dagnall used a standard bilateral eye movement design with auditorily presented stimuli, and they *did* replicate the SIRE effect for words on a recognition test [7]. Save for different word lists, there are no major methodological differences between Parker and Dagnall’s experiment and our own (including the presentation rate of words) [7]. We then thought that perhaps the bilateral stimulation of eye movements needed to occur within the same modality as the stimulus presentation (as was the case in previous studies using bilateral eye movements and visual presentation of words). Upon further investigation, however, we discovered an insightful article by Nieuwenhuis et al., which used visually presented stimuli but which had a condition where bilateral stimulation was provided via tactile sensation (alternating tapping of the participants’ hands) and a SIRE effect was observed [73]. Thus, we see no obvious reason for our failure to find a significant SIRE effect in this experiment.
